# Surface-ligand effect on radiosensitization of ultrasmall luminescent gold nanoparticles

**DOI:** 10.1142/S1793545816420037

**Published:** 2016-05-13

**Authors:** Xingya Jiang, Bujie Du, Mengxiao Yu, Xun Jia, Jie Zheng

**Affiliations:** * Department of Chemistry and Biochemistry, The University of Texas at Dallas, 800 W. Campbell Rd., Richardson, TX 75080, USA; † Department of Radiation Oncology, The University of Texas Southwestern Medical Center, 5323 Harry Hines Boulevard, Dallas, TX 75390, USA

**Keywords:** Gold nanoparticles, surface-ligand, cell uptake, radiosensitizer, radiation-protecting

## Abstract

Gold nanoparticles (AuNPs) could serve as potential radiotherapy sensitizers because of their exceptional biocompatibility and high-Z material nature; however, since *in vitro* and *in vivo* behaviors of AuNPs are determined not only by their particle size but also by their surface chemistries, whether surface ligands can affect their radiosensitization has seldom been investigated in the radiosensitization of AuNPs. By conducting head-to-head comparison on radiosensitization of two kinds of ultrasmall (~2 nm) near-infrared (NIR) emitting AuNPs that are coated with zwitterionic glutathione and neutral polyethylene glycol (PEG) ligands, respectively, we found that zwitterionic glutathione coated AuNPs (GS-AuNPs) can reduce survival rates of MCF-7 cells under irradiation of clinically used megavoltage photon beam at low dosage of ~2.25 Gy. On the other hand, PEG-AuNPs can serve as a radiation-protecting agent and enabled MCF-7 cells more resistant to the irradiation, clearly indicating the key role of surface chemistry in radiosensitization of AuNPs. More detailed studies suggested that such difference was independent of cellular uptake and its efficiency, but might be related to the ligand-induced difference in photoelectron generation and/or interactions between AuNPs and X-ray triggered reactive oxygen species (ROS).

## 1. Introduction

Radiation therapy, using high-energy ionizing radiation to damage the DNA of cancerous cells, has been a major cancer treatment in the clinical practices.^1,2^ However, ionizing radiation itself cannot differentiate malignant tissues from healthy ones. As a result, substantial side effects resulted from the damage of healthy tissues and organs have been a long-term challenge in radiotherapy.^3^ To address this challenge, radiosensitizers that can enhance the selectivity of ionizing radiation to cancerous tissues and decrease the required radiation doses are highly desired and have been extensively investigated. For instance, silicon nanoparticles,^4^ germanium nanoparticles,^5^ platinum-based compounds^6,7^ and semiconductor quantum dots^8^ were all reported to be potential radiosensitizers. In particular, gold is relatively bioinert and has a large atomic number (Z = 79), which means it has a larger photoelectric absorption cross-section (larger atom radius) and potential stronger radiation enhancement compared with other smaller Z material like carbon. Thus gold nanoparticles (AuNPs) have been extensively studied for radiosensitizer applications.^9^ Zhang *et al.* investigated the radiosensitization effect of different-sized (4.8–46.6 nm) PEG-coated AuNPs *in vitro* and *in vivo* under 662 KeV photon radiation and found that AuNPs with core sizes of 12.1nm and 27.3nm showed high radiation enhancement.^10^ Burn *et al.* compared the radiation enhancement factors of different-sized (8–92 nm) AuNPs in DNA solutions and revealed that large AuNPs had greater radiation enhancement under 50 KeV photon radiation.^11^ While these studies have implied that the particle size of AuNPs played a key role in radiosensitization, another key structural factor, surface chemistry that has significant affect on the interactions between NPs and biological systems,^12,13^ was often ignored in these studies. Until now, it is still not clear whether the same sized AuNPs coated with different surface ligands can result in distinct radiosensitization enhancements. Herein, we report head-to-head comparison on radiosensitization effects of ultrasmall NIR-emitting AuNPs coated with two antifouling surface ligands, zwitterionic glutathione and neutral PEG, respectively. Comparing with MCF-7 cells treated only with X-ray, we found that MCF-7 cells incubated with GS-AuNPs for 2 h and 24 h had 30.4 ± 13.5% and 21.6 ± 11.5% decrease in survival rates, respectively after 2.25 Gy X-ray exposure for 30 s. On the other hand, surprisingly, MCF-7 cells incubated with PEG-AuNPs for 2 h and 24 h exhibited 97.4 ± 59.3% and 19.5 ± 25.5% higher survival rates after the same X-ray exposure. While the cell uptake study showed that the GS-AuNPs can be much more efficiently internalized by MCF-7 cells than that of the PEG-AuNPs (~20 fold difference after 24 h incubation), our further studies showed that the distinct radiosensitization behaviors of GS-AuNPs and PEG-AuNPs are independent of their cellular uptake efficiency but originated from distinct radiosensitization enhancements governed by surface ligands.

## 2. Materials and Experiments

### 2.1. Materials and equipment

HAuCl_4_·3H_2_O, L-glutathione (reduced), thiolated PEG (PEG-SH, average Mn = 800) and all the other chemicals were obtained from Sigma-Aldrich (US) and used as received. 3.5KDa dialysis tube and 6-well cell culture plate and cell culture medium (DMEM) were purchased from Fisher Scientific (US). A Varian 50 Bio UV–Vis spectrophotometer was used to acquire absorption spectra. A PTI QuantaMasterTM 30 fluorescence spectro-photometer was used to acquire luminescence spectra. TEM images were obtained by a 200 kV JEOL 2100 transmission electron microscope. Hydrodynamic diameters (HDs) of the AuNPs were measured by a Malvern particle size analyzer. The elemental analysis of gold was conducted using Agilent 7900 ICP-MS. The cell X-ray radiation experiment was performed by a clinical Vero linear accelerator with 6MV photon beam.

### 2.2. Preparation of GS-AuNPs and PEG-AuNPs

GS-AuNPs and PEG-AuNPs were prepared by a thermal reduction method that previously reported by our group.^14^ Briefly, 5mL 24mM ligand (Glutathione or PEG-SH) solution was mixed with 45mL DI water at 95°C, then followed by adding 150 uL 1M HAuCl_4_ solution under stirring. The mixture was then continued to react at 95°C until the luminescence intensity of the mixture reached its maximum. After the reaction, for GS-AuNPs, ethanol was added to precipitate the GS-AuNPs, then the pellet was redispersed in DI water and centrifuged at 21,000 g to remove large aggregates, and the NIR-emitting GS-AuNPs collected from supernatant were freeze-dried for further usage. For PEG-AuNPs, the mixture was first freeze-dried and then redispersed in DI water (or PBS) and centrifuged at 21,000 g to remove large aggregates, NAP-25 column was used to purify PEG-AuNPs before usage.

### 2.3. In vitro radiation experiment

All cell medium used for cell culture contained 10% fetal bovine serum and 1% penicillin–streptomycin. The clonogenic assay was used to evaluate the radiotherapy effect *in vitro*. To evaluate sole X-ray radiotherapy effect, human breast cancer MCF-7 cells were seeded 300 cells/well in each well of five 6-well culture plates (*n* = 6) and incubated in cell incubator for 12 h, after the cells attached to cell plate surface, these cell plates were exposed to 0 Gy (no X-ray exposure), 2.25 Gy, 4.5 Gy, 6.75 Gy and 9 Gy X-ray radiation at a rate of 4.5 Gy/min respectively. After radiation, these cell pates were incubated in cell incubator for one week to allow the formation of cell colonies. Then cell medium was removed from each well and a 1mL mixture of 6% formaldehyde and 0.05% crystal violet was added into each well of the cell culture plates under room temperature to fix and stain the cell colonies. After 20 min, the formaldehyde and crystal violet mixture was carefully removed from cell plates and each well was washed twice with DI water and air-dried at room temperature. The resulting stained cell colonies were taken pictures and counted by ImageJ software. To evaluate the radiosensitization effect of AuNPs under low dose radiation condition, MCF-7 cells were plated in each well of eight 6-well cell culture plates (*n* = 6) at a density of 600 cells/well. These eight 6-well plates were divided into two groups: the first group contains five plates and the second group contains the rest three plates. Cell culture plates in the first group were treated respectively with 1uM GS-AuNPs for 2 h and 24 h, 1uM PEG-AuNPs for 2 h and 24 h, and cell medium only for 24 h. Cell culture plates in the second group were treated respectively with 1uM GS-AuNPs, 1uM PEG-AuNPs and cell medium only for 24 h. Then cell culture plates in the first group were exposed to 2.25 Gy X-ray radiation at a rate of 4.5 Gy/min and cell culture plates in the second group were used as control (no X-ray exposure). After radiation, the cell medium in all the cell culture plates were removed and PBS were used to wash each well twice before fresh cell medium were implemented. Then, the eight cell culture plates were incubated for one week to allow colony growth and cell colonies were stained and counted the same way as stated above. Since the number of cells seeded remains constant for each experiment, the resulting cell surviving fraction is calculated as (number of colonies formed after X-ray treatment)/( number of colonies formed before X-ray treatment).

### 2.4. GS-AuNPs and PEG-AuNPs cell uptake experiment

MCF-7 cells were plated in each well of four 6-well cell culture plates (*n* = 6) at a density of 10^5^ cells/well. All cell plates were incubated 24 h for cell attachment. Then half of the cell plates were treated with 1 uM GS-AuNPs and the other half with 1 uM PEG-AuNPs for certain time periods (2 h and 24 h for both AuNPs). At each time point, cell medium was removed and washed carefully with PBS three times to remove any uninternalized AuNPs, and then cells in each well were trypsinized and collected in separate plastic tubes. The cell number in each tube was counted by a hemocytometer and the AuNPs amount was quantified by ICP-MS.

## 3. Results and Discussion

### 3.1. Synthesis and characterization

**Fig. 1:**
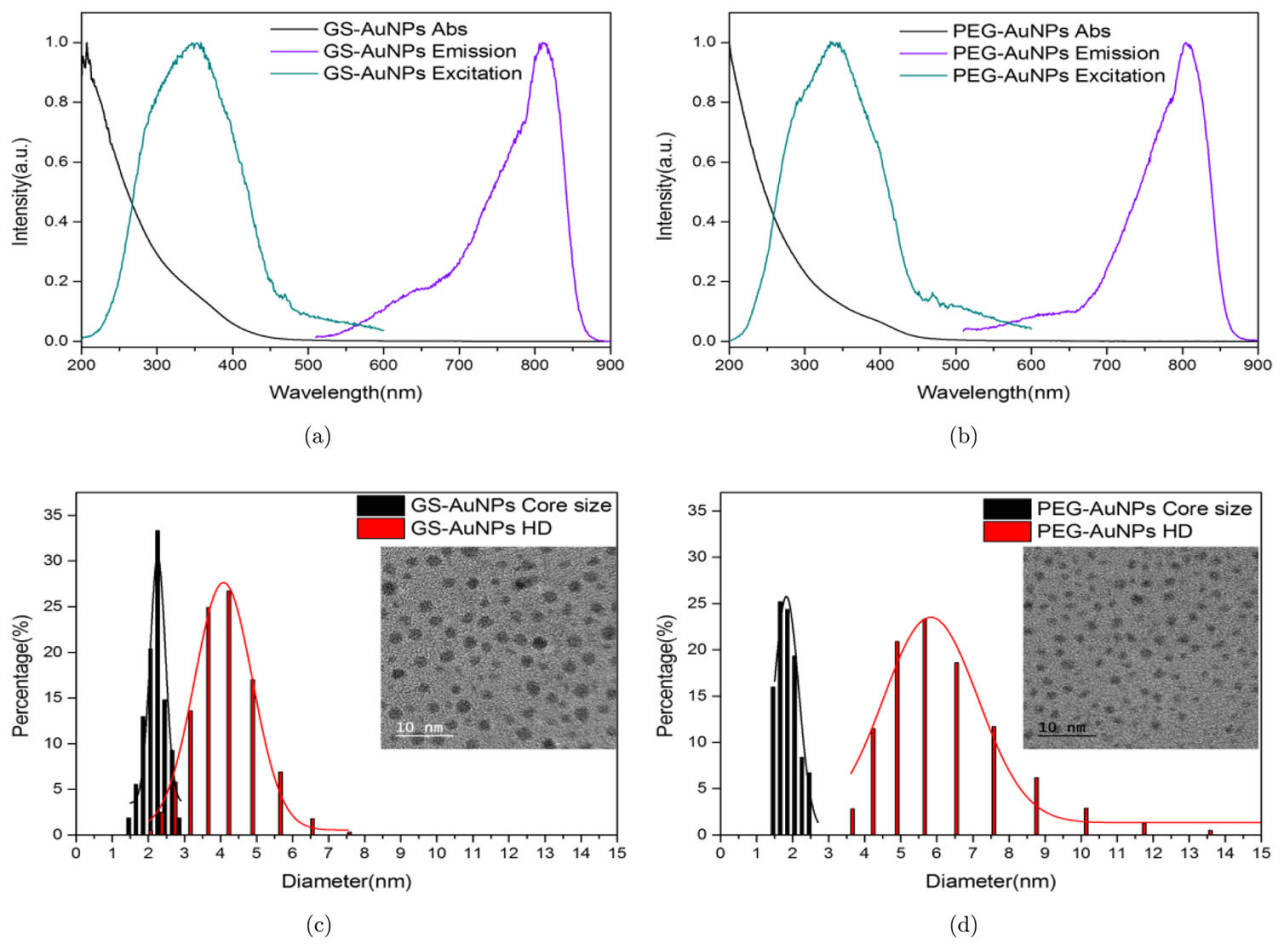
Characterizations of GS-AuNPs and PEG-AuNPs. Normalized absorption, excitation and emission spectra of GS-AuNPs, (a) and PEG-AuNPs, (b) Core size and HD of GS-AuNPs, (c) and PEG-AuNPs (d); inset is the corresponding AuNPs TEM image.

GS-AuNPs and PEG-AuNPs were synthesized by a facial thermal reduction method and their core sizes, HDs and photophysical properties were characterized (see Fig. 1). The GS-AuNPs have a core size of 2.26 ± 0.25nm and HD of 4.08 ± 0.95nm in PBS; the PEG-AuNPs have a core size of 1.82 ± 0.34nm and HD of 5.83 ± 1.31nm in PBS. Both of the AuNPs are too small to support surface plasmon resonance and they all show exponential decay profile absorption. Moreover, both AuNPs have NIR emission, which enables them to be used in bioimaging field.^15,16^ The large stokes shifts of the spectra indicate that the NIR emissions originate from ligand-to-metal charge transfer instead of transitions within the gold core. It is noteworthy that both the zwitterionic glutathione ligand and amphiphilic PEG ligand are frequently used ligands to minimize serum protein binding and increase the biocompatibility of nanoparticles. Therefore, with very similar core sizes, the GS-AuNPs and PEG-AuNPs provide us a good opportunity to evaluate the effect of two popular ligands on ultrasmall AuNPs radiosensitization.

### 3.2. In vitro radiosensitization study

**Fig. 2:**
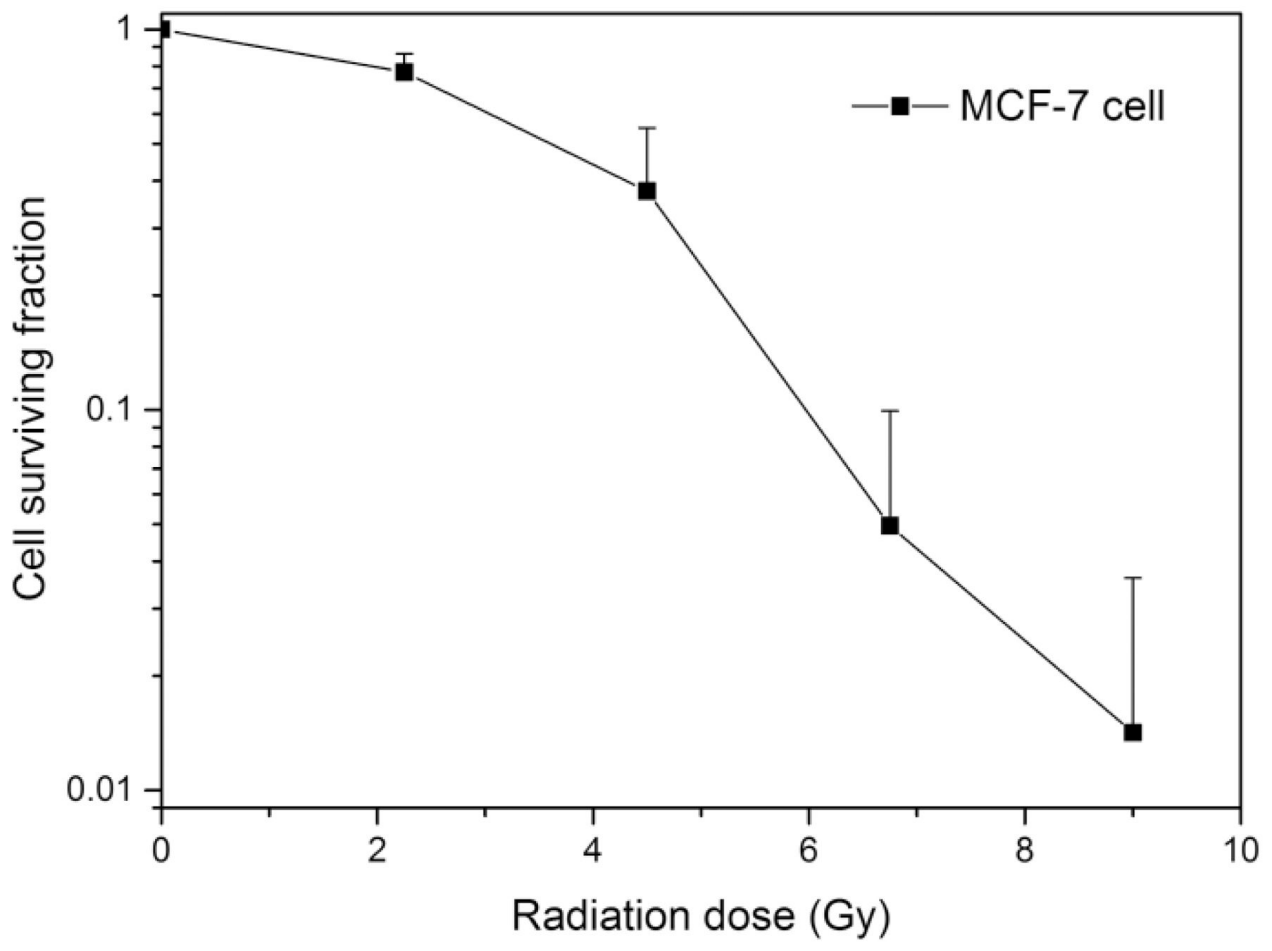
Radiotherapy effect of MCF-7 cells.

**Fig. 3:**
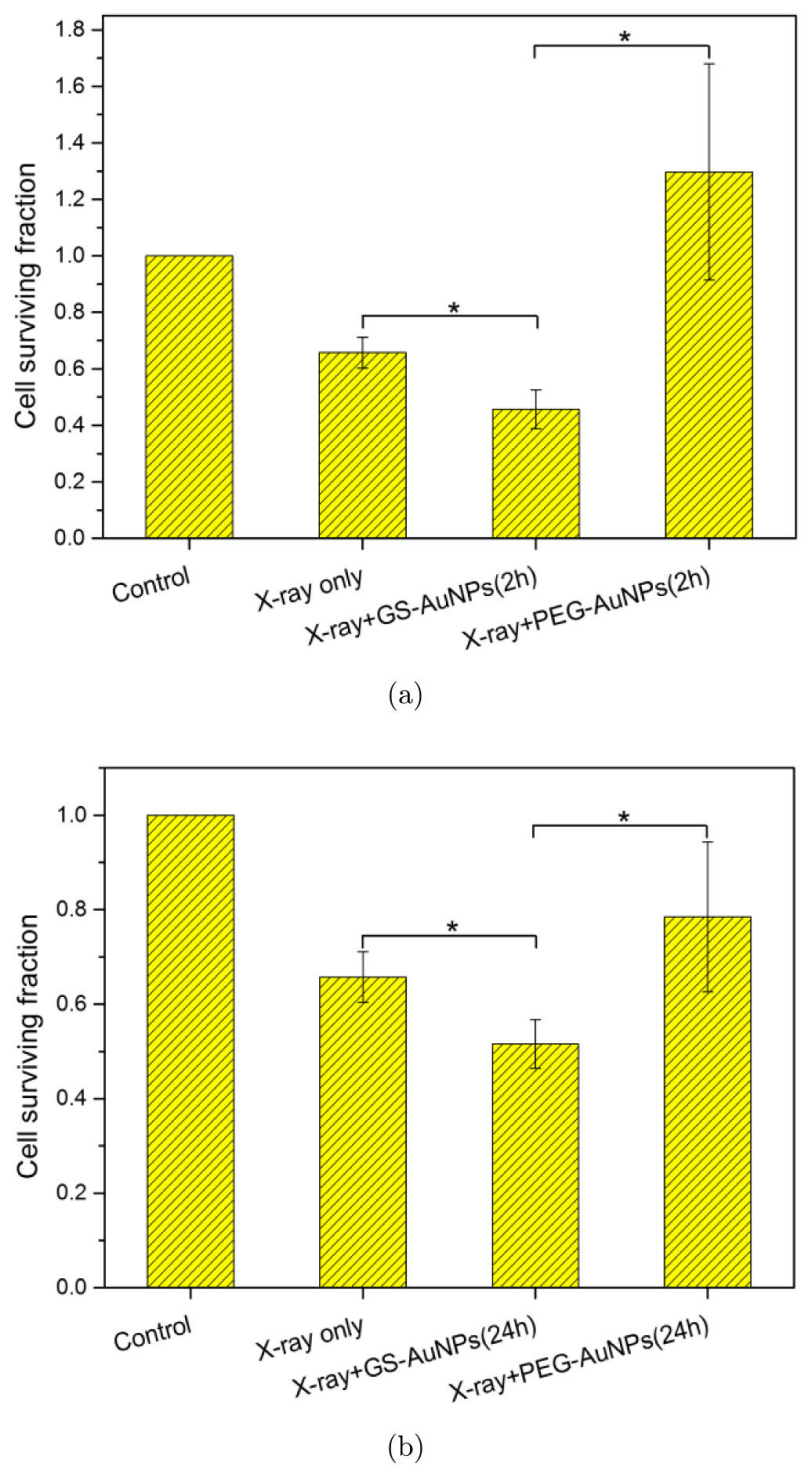
*In vitro* radiosensitization effect of GS-AuNPs and PEG-AuNPs after 2 h incubation, (a) and 24 h incubation, (b) X-ray dose is 2.25 Gy for both studies. *P < 0.05 by two-sample *t*-test.

**Scheme 1:**
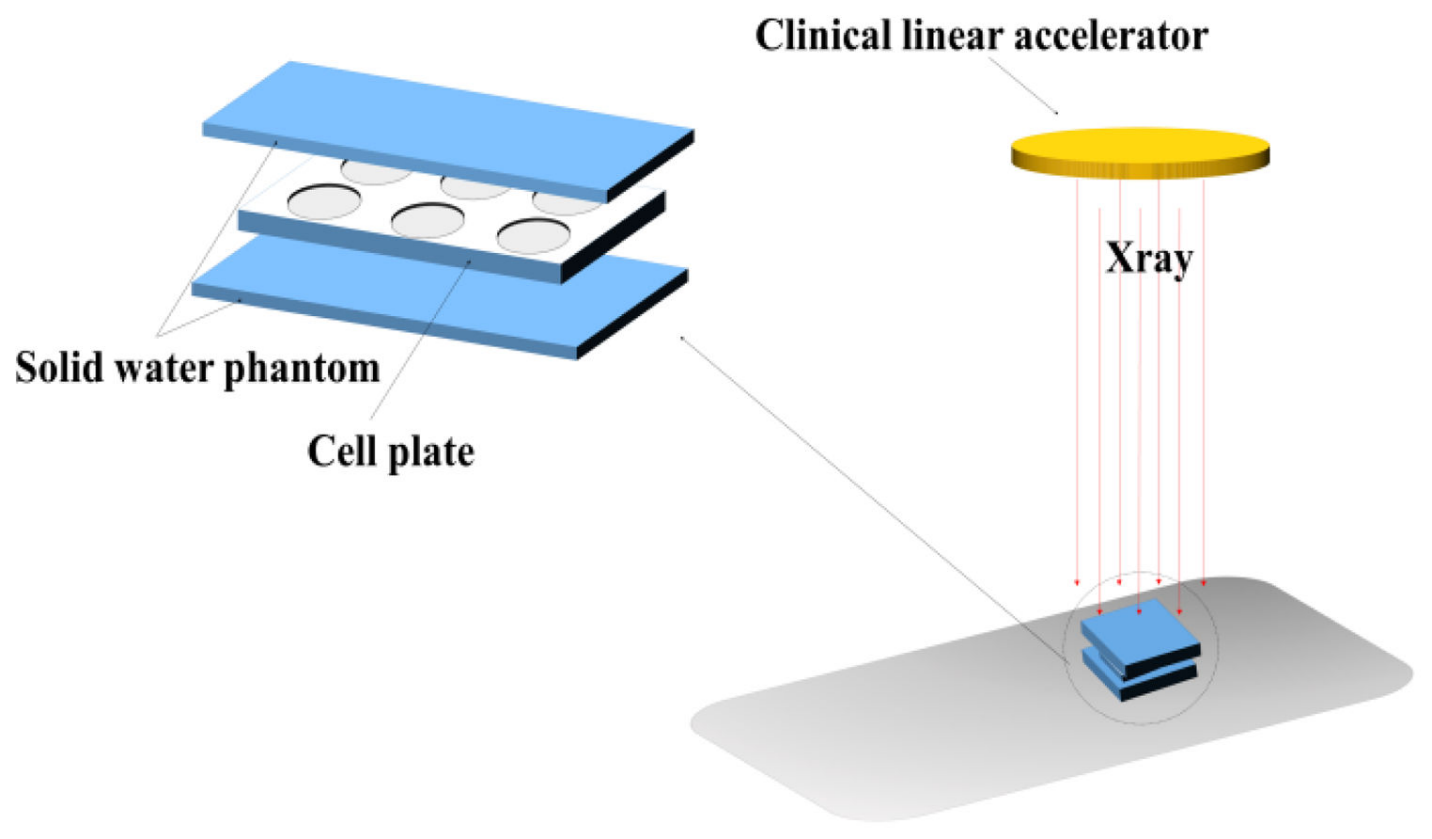
*In vitro* radiation experiment setup.

Various radiation sources with different beam energies have been reported to study the radiotherapy enhancement effect of AuNPs.^17–19^ Compared to typical human tissue, gold has bigger photoelectric cross-section at low photon energy regime (ortho-voltage range). Its radiosensitization effect may be more pronounced in this range. However, such lower energy radiation mainly deposit its dose upon entry, namely skin, and will cause severe skin burn.^1^ Apart from treating superficial cancers, nowadays most clinical radiotherapy machines use megavoltage (6–25MV) radiation beams, which have deeper penetration depth as well as less interaction with skin and can be used to deliver and focus most of the radiation dose to the deep-seated tumors. To investigate the radiosensitization effect in a clinically relevant setting, a clinical X-ray linear accelerator with 6MV beam energy was chosen to perform the *in vitro* radiation study and solid water phantoms were used to calculate the dose delivered to the cells. The radiation experiment setup is shown in Scheme 1. In order to verify the feasibility of this experiment setup, human breast cancer MCF-7 cells (without AuNPs) were first irradiated by different doses of X-ray and the radiotherapy effect was evaluated by clonogenic assay (see Fig. 2). With increase of radiation dosage, the cell surviving fraction dropped significantly, particularly at higher dosage, comparable with previously reported MCF-7 cells radiotherapy results in the same range.^20^ GS-AuNPs and PEG-AuNPs *in vitro* radiosensitization effects were also evaluated under the same conditions. Since the purpose of using radiosensitizers is to reduce the radiation dose required for efficient cancer cell killing and minimizing radiotherapy side effects, both the AuNPs radiosensitization studies were carried out under a radiation dose of ~2.25 Gy. Figure 3 shows the radiosensitization results of the GS-AuNPs and PEG-AuNPs. For MCF-7 cells incubated 2 h with AuNPs (Fig. 3(a)), comparing with X-ray only, cells with GS-AuNPs exhibited a 30.4 ± 13.5% less cell surviving rate after radiation while cells incubated with PEG-AuNPs showed a surprising 97.4 ± 59.3% increase in cell surviving rate. After 24 h incubation, similar results were still observed that PEG-AuNPs increased the cell surviving rate by 19.5 ± 25.5% while a 21.6 ± 11.5% more cell death was achieved for cells with GS-AuNPs (Fig. 3(b)). These results clearly indicated that the surface ligands of these ultrasmall AuNPs indeed played an important role in their radiosensitization behaviors.

### 3.3. AuNPs cell uptake study

**Fig. 4:**
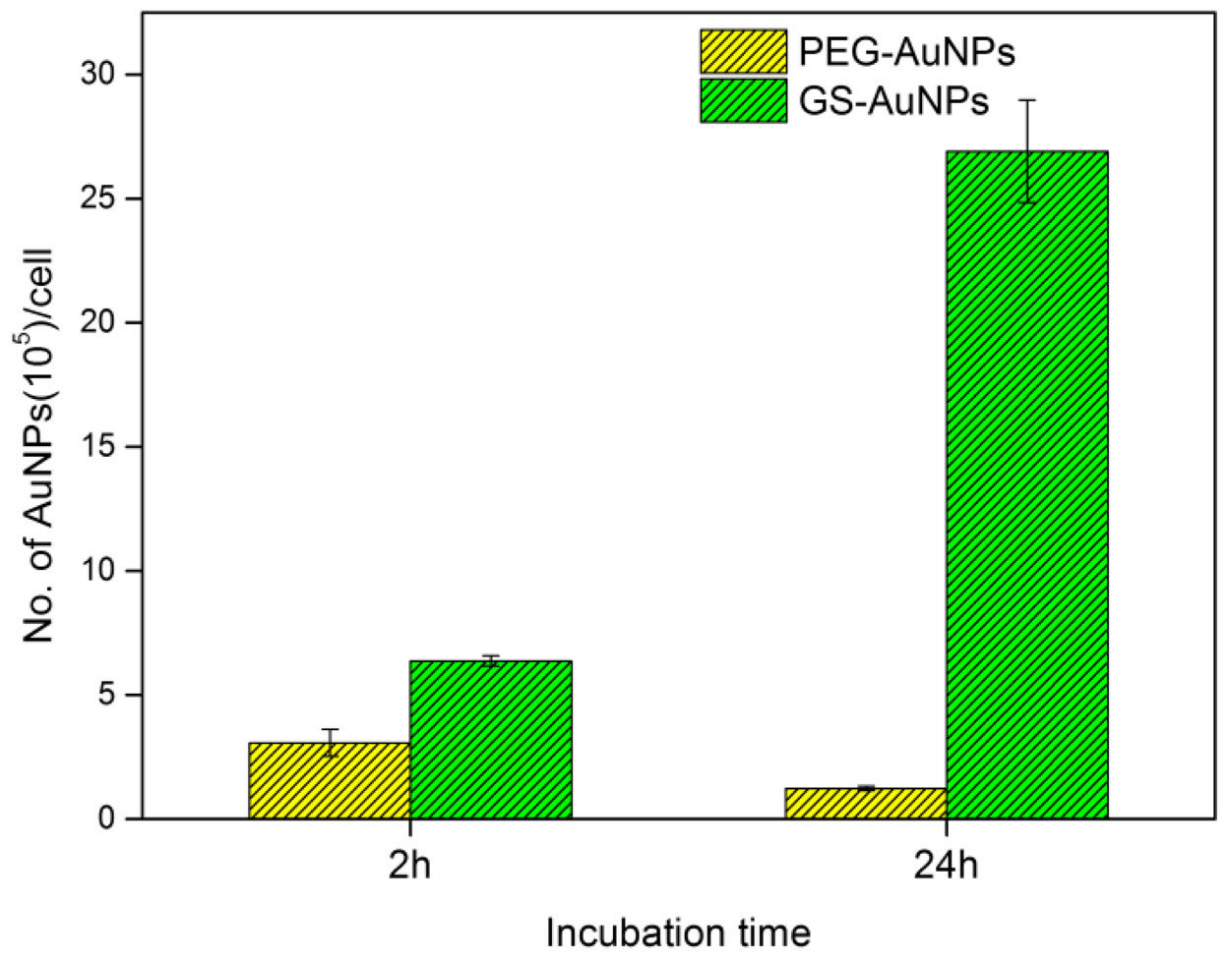
MCF-7 cells uptake of PEG-AuNPs and GS-AuNPs after 2 h and 24 h incubation.

To determine whether the observed difference in radiosensitization between GS-AuNPs and PEG-AuNPs resulted from different cell uptake, we investigated the MCF-7 cells uptake efficiency of GS-AuNPs and PEG-AuNPs after 2 h and 24 h incubation by ICP-MS. As shown in Fig. 4, GS-AuNPs were internalized much more efficiently than that of PEG-AuNPs, nearly a ~20 fold cell uptake difference at 24 h time point in terms of the number of AuNPs per cell, which might be due to specific glutathione receptors on the cell membranes. However, further comparison on radiosensitization of GS-AuNPs showed a slight increase (12.9 ± 18.9%) in cell surviving rate from 2 h to 24 h incubation even though the amount of GS-AuNPs internalized by MCF-7 cells was increased 321.5 ± 34.6%. These results clearly suggested the cellular internalization of GS-AuNPs did not contribute to the radiation enhancement significantly. Moreover, at 2 h incubation time point, the ratio of internalized GS-AuNPs and PEG-AuNPs was ~2 and the ratio of cell surviving rate was ~2.8 (PEG-AuNPs: GS-AuNPs); on the other hand, at 24 h time point, the ratio of AuNPs internalized was ~20, which was 10 times larger than that of 2 h time point, but the ratio of cell surviving rate decreased to ~1.5. These results further indicated that the different radiosensitization behaviors of GS-AuNPs and PEG-AuNPs were uncorrelated with their cell uptake efficiency. It has been reported that surface ligands have certain impact on the photoelectrons ejected from nanoparticles.^21,22^ Besides, X-ray triggered ROS have been known to play an important role in cell radiation damage, and the surface chemistry effects on ROS generation were also reported.^23,24^ Therefore, we hypothesize that the observed surface-ligand effect on radiosensitization very likely originated from the differences in X-ray induced photoelectron generation and/or interactions between AuNPs and X-ray triggered ROS.

## 4. Conclusion

By conducting head-to-head comparison on radiosensitization of AuNPs with very similar sizes but different surface chemistries at *in vitro* level, we found that zwitterionic GSH and neutral PEG ligands have distinct radiosensitization effect. GS-AuNPs can enhance X-ray therapeutic efficacy by 30.4 ± 13.5% but PEG-AuNPs significantly reduce X-ray therapeutic efficacy by 97.4 ± 59.3% at low X-ray dosage under the clinically used irradiation energy of 6MV photon beam. While the underlying mechanisms behind this phenomenon are still unclear, these results clearly indicate that surface chemistries do have distinct impacts on radiotherapy of AuNPs and can turn a radiosensitizer into a radiation-protecting agent. Little correlation between radiosensitization and AuNPs cellular uptake efficiency suggested that radiation efficacy is not dependent of how much AuNPs accumulated inside cells but probably more strongly depends on the photoelectrons ejected by AuNPs and/or interactions between AuNPs and X-ray triggered ROS, though all processes are affected by surface ligands. Further study is needed to unravel the mechanisms of this ligand-induced difference in radiosensitization. These studies offer an understanding of surface-ligand effect on radiosensitization, which is expected to expedite the development of more potent theranostics for both imaging and therapy as well as a new generation of radiation protecting agents.
